# Concentration of Pro-Health Compound of Sorghum Grain-Based Foods

**DOI:** 10.3390/foods11020216

**Published:** 2022-01-13

**Authors:** Jakub Frankowski, Anna Przybylska-Balcerek, Kinga Stuper-Szablewska

**Affiliations:** 1 Department of Bioeconomy, Institute of Natural Fibres & Medicinal Plants-National Research Institute, ul. Wojska Polskiego 71b, 60-630 Poznań, Poland; jakub.frankowski@iwnirz.pl; 2 The Department of Chemistry, Faculty of Forestry and Wood Technology, Poznań University of Life Sciences, ul. Wojska Polskiego 75, 60-101 Poznań, Poland; kinga.stuper@up.poznan.pl

**Keywords:** bioactive compounds, phenolic compounds, phenolic acids, flavonoids, phytosterols, sorghum, functional food

## Abstract

More than 35% of the world sorghum seed production is a human food source. The main ingredient of fully ripe sorghum grains is starch. Sorghum does not contain gluten, and it is also a rich source of antioxidant compounds other than vitamins or macro- and microelements, including phenolic acids, flavonoids, and sterols. The aim of this study was to determine the antioxidant activity and the content of selected bioactive compounds, i.e., total phenolic acids, total flavonoids, and total phytosterols, as well as determination of the qualitative and quantitative profile of phenolic acids, flavonoids, and phytosterols in various food products, the basic ingredient of which was sorghum grain. It was found that antioxidant activity is related to the total phenolic compounds content. The ABTS^•+^ ranged from 319 to 885 µmol TROLOX/kg. However, white sorghum grain flour contained almost two times more polyphenols than red sorghum grain flour. The FPA ranged from 224 in raw pasta to 689 mgGAE/100 g in white sorghum grain. During this study, the quantitative profile of selected polyphenols in grain flour, wafers, pasta, and cookies containing sorghum grain was also investigated, as well as the content of 11 selected phenolic acids. Total content of the latter ranged from 445 to 2850 mg/kg. Phytosterols such as beta-sitosterol, campesterol, and stigmasterol were found in all the analyzed products. Based on this research, it was investigated that the products containing sorghum grains can be classified as functional food.

## 1. Introduction

Sorghum, as a valuable nutritional plant, is primarily a source of food for humans [1,2,3,4,5] and a feed for animals [6,7]. Therefore, over 35% of the world production of sorghum seeds (estimated at over 60 million tons per year) is the basic nutritional product [2,8,9]. The main ingredient of fully ripe sorghum grains is starch. Depending on the variety, 100 g of grains contain 65 to 80 g of it. Apart from that, the seeds are also a source of proteins (7–15 g), other polysaccharides (up to 10 g), and lipids (1.5–6 g) [2,10,11]. The content of chemical compounds in sorghum grain is similar to maize and other cereals. Basic composition testing showed that 10 significant differences in the starch content were found between the sorghum cultivars, even those with different seed color [1]. Dishes prepared from sorghum varieties with a high tannin content stay in the stomach longer, increasing and prolonging the feeling of fullness [12,13,14]. They are also characterized by a low glycemic index, which is why they are recommended for people with diabetes [15,16,17,18].

About 60% of these organic compounds are caphyrins—a group of prolamines found only in these plants [18]. The sorghum grains also contain albumin, globulins, and glutelins, which (similarly to prolamines) are the main source of kernel storage proteins [19,20].

Furthermore, sorghum is a source of polyunsaturated fatty acids f.eg.: linoleic (49%), oleic (31%), palmitic (14%), linolenic (2.7%), and stearic (2.1%) [21,22]. Sorghum is also a good source of vitamins, especially those from the B group and fat-soluble vitamins (A, D, E, and K) [2,23].

Sorghum can be used to prepare gluten-free products so as to fulfill the needs of people with celiac disease [24,25,26,27]. Its grain can be successfully used to prepare gluten-free pasta. All the tested mixtures demonstrated nutritional value and represent a good potential alternative to current commercial pasta [28,29,30,31,32,33,34].

Additionally, sorghum is a rich source of antioxidant compounds such as vitamins or macro- and microelements, including phenolic acids, flavonoids, and sterols [35,36,37,38,39,40]. Each of these groups of substances has a specific effect on the human body and is involved in various physiological processes. They prevent the development of civilization diseases, such as diabetes, caries, and obesity, and support the functioning of the cardiovascular, nervous, and immune systems. They are also the build components of tissues and parts of hormones and enzymes. Moreover, these substances regulate the hormonal balance, support metabolic processes, facilitate the absorption of nutrients, and inhibit inflammatory diseases and allergies. They are involved in the transformation of fat and cholesterol, support the proper peristalsis of the intestines, and protect against cancer. All the described ingredients shape the quality of potential sorghum processing products. The resulting food products can be classified as so-called functional foods. Important factors influencing the level of bioactive compounds are application forms, cultivars, and growing conditions (agrotechnical and meteorological) [35]. The research [35,36,37,38,41] carried out so far has focused on the analysis of a raw material such as sorghum grain. The expansion of the market offer of products containing sorghum, such as flours, groats, cookies, wafers, bread, and pasta, contributed to the start of the research described in this article. The aim of the study was to assess the content of selected bioactive compounds and the antioxidant potential of available food products containing sorghum grain.

## 2. Materials and Methods

The tested food products were purchased in a chain of retail stores in Poland in 2020. The composition of the tested food products is presented in Table 1.

As part of this study, the series of analyses were undertaken to determine the antioxidant activity and the content of selected bioactive compounds, i.e., total phenolic acids, total flavonoids, and total phytosterols, as well as determination of the qualitative and quantitative profile of phenolic acids, flavonoids, and phytosterols in food products whose basic ingredient was grain sorghum.

For chemical analyzes, samples of nutritional products weighing 100 g were taken. The sorghum products were ground in a WZ-40 laboratory mill.

### 2.1. Extraction of Phenolic Compounds and Their Chromatographic Analysis

Samples for analysis, each weighing 0.20 g, were prepared. Then, they were placed in 20 mL culture tubes, in which two hydrolyses, such as basic, followed by acid hydrolysis, were carried out. The alkaline hydrolysis consisted of adding 1 mL of distilled water and 4 mL of 2M aqueous sodium hydroxide solution to the test material placed in culture tubes. The samples prepared in this way were closed with a stopper and then subjected to prolonged heating in a water bath at 95 °C for half an hour. In the next step, the test tubes with samples were cooled to room temperature, around 21 °C, and then, the contents of the tubes were neutralized with 2 mL of 6M aqueous hydrochloric acid solution (pH = 2). The neutralization process was carried out in ice water each time. Extraction of selected polyphenols from the inorganic phase was carried out twice using 2 mL of diethyl ether. The resulting ether extracts were transferred to 10 mL vials. In the next step, a second hydrolysis such as acid hydrolysis was performed. For this, the aqueous phase remaining after the first step was supplemented with 3 mL of a 6M aqueous hydrochloric acid solution. The samples prepared in this way were closed with a stopper and then subjected to prolonged heating in a water bath at 95 °C for half an hour. In the next step, the sample tubes were cooled to room temperature, around 21 °C. Extraction of selected polyphenols from the inorganic phase was carried out twice using 2 mL of diethyl ether. The resulting ether extracts were transferred to 10 mL vials and then evaporated to dryness under a nitrogen stream in a RapidVac evaporator. Immediately before the chromatographic analysis, the samples were dissolved in 1 mL of methanol and ultrasonic waves. Samples dissolved in this way were cleaned on a syringe filter. In the next stage, the chromatographic analysis was carried out using the Aquity class H UPLC (Ultra High Performance/Pressure Liquid Chromatography) system with a Waters Acquity PDA detector (Waters, Milford, MA, USA). The chromatographic separation was performed on an Acquity UPLC^®^ BEH C18 column (100 mm × 2.1 mm, particle size 1.7 µm) (Waters, Ireland). The elution was carried out by gradient using the following mobile phase composition: A: acetonitrile with 0.1% formic acid, B: 1% aqueous formic acid mixture (pH = 2). The concentrations of polyphenols were determined using an internal standard at a wavelength of λ = 320 nm (flavonoids) and λ = 280 nm (phenolic acids). Polyphenols were identified by comparing the retention times of the analyzed peak with the retention time of the standard and by adding a specific amount of the standard to the analyzed samples and repeating the analysis. The detection level is 1 µg/g. [38].

### 2.2. Spectrophotometric Analysis Total Polyphenolic Contents

For the analysis of total polyphenols, samples weighing 100 g were prepared. In order to better isolate phenolic compounds, the samples were ground in a laboratory mill (WŻ-1). For proper analysis, samples weighing 10 g were prepared. To determine the total content of phenolic compounds, the prepared samples were extracted with 80% methanol. The fragmented test material was poured into 100 mL of 80% methanol and then placed in an ultrasonic bath for half an hour. At this time, the precipitate was collected in round bottom distillation flasks. The extraction process was repeated three times. In the next step, the combined extracts were evaporated to dryness on a rotary evaporator. The phenolic compounds were quantitatively transferred to a vial with methanol and dried under a stream of nitrogen in a RapidVac evaporator. For sample preparation for specific analysis, 0.5 mL of deionized water and 0.125 mL of Folin–Ciocalteu reagent (Fluka) were added to 0.125 mL of extract, and after 6 min, the mixture was supplemented with 1.25 mL of 7% aqueous Na_2_CO_3_ solution and 1 mL of deionized water. Then, after 1.5 h, spectrophotometric analysis was performed using the Helios Thermo Electron Corp spectrophotometer. In this case, the absorbance was read at λ = 760 nm in relation to water. The results are expressed as mg gallic acid/kg DM. sample [38].

### 2.3. Chromatographic Analysis of Phytosterols

For the analysis of total phytosterols, samples weighing 100 g were prepared. In order to better isolate phytosterols, the samples were ground in a laboratory mill (WŻ-1). For the proper analysis, samples weighing 0.1 g were prepared. Samples containing 0.1 mg of ground material for testing were placed in 20 mL culture tubes. Then, 2 mL of methanol and 0.5 mL of 2M aqueous sodium hydroxide solution was added. The samples prepared in this way were tightly closed with a stopper in test tubes. The culture tubes were then placed in 300 mL plastic containers, which were sealed and placed in a microwave oven (Model AVM 401/1WH, Whirlpool, Sweden) operating at 2450 MHz and 900 W. The samples were subjected to microwave waves for 20 s and 370 watts, cooled to room temperature around 21 °C, and then the microwave wave treatment was repeated. The samples were cooled to room temperature, around 21 °C, and then neutralized with 1M aqueous hydrochloric acid solution; 2 mL of methanol was added and to isolate the selected sterols, and extraction was carried out three times with pentane, 4 mL each time. The combined pentane extracts were evaporated to dryness under a nitrogen stream in a RapidVac evaporator. Prior to actual analysis, the samples were dissolved in 4 mL of methanol and, for purification prior to chromatographic analysis, filtered using syringe filters with a pore diameter of 0.5 mm (Fluoropore Membrane Filters, Millipore, county cork, Ireland). The sample extract was analyzed on an Aquity class H UPLC (Ultra High Performance/Pressure Liquid Chromatography) system equipped with a Waters Acquity PDA detector (Waters, USA). Chromatographic separation was performed on an Acquity UPLC^®^ BEH C18 column (100 mm × 2.1 mm, particle size 1.7 μm) (Waters, Dublin, Ireland) eluting with methanol/acetonitrile/water (85:10:5) at a flow rate of 0.4 mL/min. Total sterols were detected with a Waters Acquity PDA detector (Waters, USA) set at 282 nm [38].

### 2.4. ABTS^•^^+^ Method

For ABTS^•^^+^ generation from ABTS salt, 3 mM of K_2_S_2_O_8_ was reacted with 8 mM ABTS salt in distilled, deionized water for 16 h at room temperature in the dark. The ABTS^•^^+^ solution was then diluted with pH 7.4 phosphate-buffered solution containing 150 mM NaCl (PBS) to obtain an initial absorbance of 1.5 at 730 nm. Fresh ABTS^•^^+^ solution was prepared for each analysis. Reaction kinetics were determined over a 2 h period with readings every 15 min. Reactions were complete in 30 min. Samples and standards (100 ím) were reacted with the ABTS^•^^+^ solution (2900 µm) for 30 min. Trolox was used as a standard. Results were expressed in ABTS^•^^+^ (µmolTROLOX/kg) d.m. sample [42].

## 3. Results and Discussion

**Figure 1:**
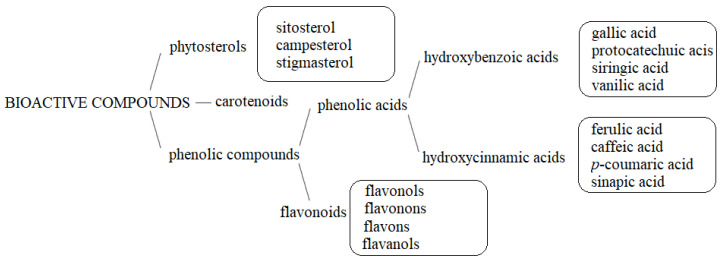
Bioactive compounds in functional food based on sorghum grains.

Consuming functional food based on sorghum grains has a positive effect on the health of the consumer. During this study of functional foods based on sorghum grain, the presence of natural bioactive ingredients was found (Figure 1). They have a beneficial effect on the health condition of the body in addition to nutritional effects.

The antioxidant activity of analyzed products was related to the total phenolic compounds content. The polyphenolic composition was mainly responsible for the high antioxidant activity of the bioactive compounds in the sorghum grain [35,42]. Based on these studies, it was found that the antioxidant activity of phenolic compounds in food products was varied (Table 2). Both white and red sorghum flours had the highest antioxidant activity. The literature on the subject reports that black and brown sorghum grains, especially its whole grain products and bran in which polyphenols are concentrated, are characterized by high antioxidant activity (ORAC = 3.124 µmol TE/g, ABTS^•+^ = 768 µmol TE/g and DPPH 716 µmol TE/g) [42,43,44,45,46]. Granati et al. and Liu and Finley noted that in vitro antioxidant activity did not reflect the actual antioxidant capacity and health benefits in vivo, as it does not take into account technological processes (i.e., regrind, temperature) or physiological conditions (i.e., pH, temperature, bioavailability and metabolism) [47,48,49]. Alferi et al. and Flores-Naveda et al. noted, in the framework of a preliminary characterization of sorghum breeding lines as potential raw materials for the development of gluten-free food products, that the content of antioxidant compounds and total antioxidant capacity were different in a lot genotypes, in depending for their grain color (ranging from white-yellow to red, brown, and black). Furthermore, correlation coefficients between antioxidant compounds and color parameters indicated that the pericarp color is not efficient marker in the selection of genotypes with specific antioxidant capacity [50,51]. Chiremba et al. noted in their experiment that total phenolic content and antioxidant activity of the cookies was generally lower than that of flours. It would seem that cookie making resulted in changes in total phenolic content and resultant antioxidant activity. However, when the flour component in the cookie is considered (57% sorghum flour in formulation), the phenolic content and antioxidant activity of cookies was slightly higher than that of flours factors believed to contribute to excess antioxidant activity include Maillard reaction products formed during high temperature that exhibit antioxidant activity and the release of bound phenolic acids from cell walls during baking [52,53].

The correlation analysis was performed and correlation coefficients were determined at the significance level of 0.95 (Table 3). The correlation between the antioxidant activity and the content of polyphenols measured by the reaction with the Folin–Ciocalteu reagent was not always significant, which was influenced by the presence of other antioxidants in the sorghum grain. The content of total polyphenols was presented in Table 2. It was found that white sorghum grain flour contains almost two times more of them than red sorghum grain flour, despite the fact that the antioxidant activity was comparable.

The differences in activity between flours were also influenced by their type. Significant differences were found between wholemeal flour and hulled grain flour. This was due to the fact that antioxidant compounds, mainly phenolic compounds and carotenoid pigments, are not bound to the cell wall and are mainly found in the pericarp and nucleus [52,54,55,56]. Chiremba et al. and Dlamini et al. found that biscuits made of various sorghum flours differ in terms of polyphenol content and antioxidant activity of the biscuits. For each variety of sorghum, cakes from 100% extraction flour had two to three times more total phenols compared to 70% extraction flours, while the antioxidant activity was 22–90% higher. The condensed tannin sorghum cookies had two to five times more phenols compared to the untanned condensed sorghum cookies. The sorghum flours had slightly higher phenolic content and antioxidant activity values than the corresponding cakes [52,54,55,56,57]. The same is true for the antioxidant activity. The next tested products were wafers, pasta, and cookies containing sorghum grain. The content of FPA in the cakes was about 25% higher than in the wafers and noodles. On the other hand, wafers had a higher antioxidant activity than those of pastries and pasta. Due to their pro-health properties, cakes made of red sorghum grain flour were as tasty as wheat-flour cakes [56,57,58]. It has been noticed that cookies with the addition of sorghum grain reduce oxidative stress and inflammation and improve the glycemic response, making them an alternative snack for people with obesity and diabetes [52,59].

During this study, the quantitative profile of selected polyphenols in food products containing sorghum grain was also investigated. Therefore, in all tested food products, the presence of selected phenolic compounds was found, i.e., 7 flavonoids (apigenin, kaempferol, luteolin, naringenin, rutin, and vitexin) and 11 phenolic acids (4-hydroxybenzoic, caffeic, chlorogenic, ferulic, *p*-coumaric, protocatechuic, sinapic, syringic, t-cinnamic, and vanillic). The literature on the subject reports that adding sorghum to pasta in an amount of 20 to 40% significantly increases the content of polyphenols and antioxidant activity [59,60,61,62]. In turn, other scientists found that it is possible to produce pasta of similar sensory quality while maintaining nutritional and health functions if its content is no more than 30% [61]. The health-promoting properties of bioactive compounds in food products with the addition of sorghum flour depend on the variety of this raw material. Khan et al. showed that eating pasta containing red sorghum grains instead of white ones, which had a lower content of phenolic compounds, significantly improved human health due to increased antioxidant activity. It was also found that pasta containing red sorghum flour (RSF) or white sorghum flour (WSF) (in amounts of 20, 30, and 40%) increased the content of bound phenolic acids, the total content of polyphenols, and the antioxidant capacity in all tested variants in compared to the control pasta (without the addition of sorghum). In turn, the content of free phenolic acids and anthocyanins was higher only in pasta containing red sorghum [61,62,63]. Xiong et al. and Ofosu et al., in their research, stated that the dominant compounds were flavonoids, with flavones being the main subclass of these three extracts. Their findings suggest that decorticated sorghum grains contain significant amounts of flavonoids and may be promising functional food additives [64,65].

The next stage of the research was the analysis of 11 selected phenolic acids. Their presence was found in all tested food products. Phenolic acids are abundant bioactive compounds in sorghum grain; depending on the cultivation region, their total content ranges from 445 to 2850 mg/kg [35,38,63]. Due to their structure and properties, phenolic acids can be divided into two categories: benzoic acid derivatives and cinnamic acid derivatives [6,35,38,66]. Phenolic acids are present mainly in the bran layer of the grain, but also in the endosperm and pericarp; therefore, whole sorghum products are a richer source of them than those from hulled grain. Phenolic acids occur both in free and bound form, which affects their bioavailability. Free phenolic acids are not bound to the cell wall and are found mainly in the pericarp and starch [54,56]. Bound phenolic acids, compared to free phenolic acids, have lower bioavailability due to the presence of covalent bonds. These bonds are broken only under the conditions of acidic or basic hydrolysis and high temperature [65]. Most of the phenolic acids (about 70 to 95%) present in the sorghum grain are bound. Among all identified phenolic acids, the highest content was found for ferulic acid, which accounts for up to 90% of all bound phenolic acids. On the basis of these studies, its presence was found in all tested products (Table 4), which is comparable to previous literature reports in which its concentration was determined at the level of 100 to 500 mg/kg in sorghum grain [35,38,66,67,68,69]. Noteworthy is also the high content of two other phenolic acids in the tested food products, i.e., *p*-coumaric acid and protocatechuic acid. Xu et al. noted that sorghum grain is being used more and more for human consumption due to its gluten-free nature and the potential health benefits of phenols. Sorghum is rich in bioactive phenolic compounds such as ferulic acid, gallic acid, and vanillic acid, which are known to provide many health benefits, including antioxidant anti-inflammatory effects. Given the growing trend in human consumption, sorghum is being used more and more often for the production of functional food [70].

The dominant flavonoids in the tested sorghum and wafer flours are: luteolin and apigenin (Table 5) [55]. In turn, in the case of pasta, it was found that luteolin was dominant, while in cookies the highest content of naringenin and kaempferol was found. Based on the available literature, it has been noticed that the content of flavones in the ground sorghum is from about 20 to 390 mg/kg [6,39,55]. Whole grain products/flours from red and yellow varieties were characterized by a high content of these substances [6,35,39,55]. Depending on the sorghum variety and technological processes, the content of these compounds varied and ranged from 0 to 2000 mg/kg [71]. Like flavones, flavanone glycosides are sensitive to low pH and are easily hydrolyzed, which makes them highly bioavailable.

**Figure 2:**
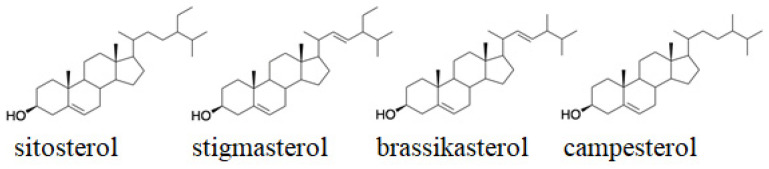
Structural formulas of selected plant sterols.

Another analyzed group of pro-health compounds were plant sterols, also known as phytosterols. Phytosterols are structurally and functionally similar compounds to cholesterol, synthesized exclusively by plants. These compounds are part of plant cell membranes, performing a function analogous to cholesterol in the membranes of animal cells, i.e., they reduce the fluidity of membranes, especially its surface layer. They are found in all plant tissues. Phytosterols are divided into three groups, the first two of which are sterols: those with a double bond between C5 and C6 (Δ5-sterols) and those with a double bond between C7 and C8 (Δ7-sterols), while the third group consists of stanols that do not exist at all, with a double bond in the molecule (campestanol, beta-sitostanol). The most important phytosterols present in cereal plants include: beta-sitosterol, stigmasterol, campesterol, alpha-5-avenasterol, and alpha-7-avenasterol (Figure 2). On the other hand, campestanol and beta-sitostanol are the dominant phytostanols. The properties of phytosterols are based primarily on the antioxidant effect, which is enhanced by the action of stressors on the plant. Therefore, they neutralize free radicals formed in plant cells as a result of a defense reaction to oxidative stress. It has been suggested that plant sterols such as β-sitosterol, campesterol, or stigmasterol may contribute to a significant reduction in LDL cholesterol levels. The research was based on the analysis of the most important phytosterols contained in sorghum grain, i.e., beta-sitosterol, campesterol, and stigmasterol (Table 6). In turn, Carr et al., on the basis of their research, found that the total content of phytosterols in ground sorghum grain is about 50 mg/100 g [72]. Similar studies were conducted by Heupel et al. claiming that the profile of dominant free sterols in sorghum seeds is campesterol, dihydrobrassicasterol, sitosterol, and stigmasterol [73]. Lee et al. analyzed the content of phytosterols (campesterol, stigmasterol, and β-sitosterol) in sorghum seeds. They found a higher content of β-sitosterol compared to campesterol and stigmasterol, and the mean content of campesterol (75.5 mg/kg) and stigmasterol (96.5 mg/kg) [74]. The phytosterol content of sorghum was similar to that of Singh et al. (2003) who reported 460 to 510 mg / kg of total phytosterols in sorghum grain, more than in maize and barley [75,76]. All these results suggest that sorghum can be used as a source of phytosterols in the human diet compared to other cereals [74,77,78].

Sorghum starch leads in carbohydrate content. The amounts of soluble sugars, pentosans, cellulose, and hemicellulose are low. Sorghum is a good source of fiber, especially the insoluble fraction. This fiber can shorten the time it takes for food to pass through the digestive system and prevent gastrointestinal problems. Protein content and composition vary with genotype, water availability, temperature, soil fertility, and environmental conditions during grain development. The protein content in sorghum is usually 11–13% [79]. The main protein fractions in sorghum are prolamines and glutelins. Characteristic for the protein composition of this grain is the lack of gluten, and its grains are low in lysine. In vitro and in vivo studies on farm and laboratory animals indicate that sorghum proteins are generally less digestible than those of other cereals [80]. The digestibility of sorghum is 74.5%, compared to 78.5% for maize [79,80]. The bioavailability of iron in sorghum is negatively affected by the presence of polyphenols and phytates. Researchers reported that the absorption of iron from sorghum beer was over 12 times greater than that from gruel [79]. This has to do with the processes the grain has had to go through. Namely, germination, malting and fermentation increase the nutritional value of sorghum by causing significant changes in its chemical composition and the elimination of anti-nutritional factors. Apart from essential macronutrients, vitamins, and minerals, the sorghum grain also contains anti-nutritional substances. They are essential for many biological processes in plants. On the other hand, they have some negative effects on the organism of humans and animals. One of the groups of anti-nutrients found in sorghum are phenolic compounds, including phenolic acids and flavonoids. Some grains contain condensed polyphenols called tannins in a layer under the seed coat, but most cultivated sorghum plants do not contain them at all. Tannins protect the grain from insects and birds, but also inhibit certain enzymes. As a result, the digestibility of the protein and the degradation of cellulose are difficult. Scientists conducted animal studies that proved that tannin inhibits protein absorption and reduces the use of minerals [79]. Feeding pigs with feed containing 4.21% tannin reduced protein digestibility by 5.6% [80]. The tannin content in unripe and dark grains is always higher than in ripe and light grains.

Sorghum also contains phytic acid, phytates that form complexes with minerals such as calcium, iron, and magnesium, making them biologically inaccessible for absorption. Studies have shown that sorghum bran contained the highest levels of phytates [80]. The last group of anti-nutritional substances are cyanogenic glycosides, the representative of which, i.e., dhurrin, is present mainly in the leaves and germinating seeds of sorghum. When germinating seeds are processed, cyanide, which is a very toxic substance, can be released. The lethal dose of dhurrin in humans is high, and sorghum contains little of it. Consequently, a person would have to eat a significant amount of raw sorghum to experience negative effects. Several studies conducted by scientists have shown that sorghum works against obesity. The results showed that the extracts of this cereal significantly inhibited the differentiation and accumulation of triglycerides [81]. Similar studies in rats showed a fairly large decrease in body weight of animals fed with feed containing sorghum. One study compared weight gain of rabbits according to the type of feed they consumed. It turned out that animals whose diets were based on maize and sorghum gained significantly less weight than those eating corn alone [81]. Human clinical trials with sorghum have also been conducted to investigate obesity-related parameters. One such study investigated the effect of sorghum biscuits on the body. It turned out that their consumption causes greater satiety compared to the participants receiving wheat [82]. Additionally, it is known that sorghum starch is digested more slowly than the starch of other cereals due to the hard outer layer of the endosperm and the presence of tannins. The results of the conducted research suggest the possibility of using sorghum as a food component that controls body weight.

## 4. Conclusions

Research on the content of selected bioactive compounds and the antioxidant activity of food products containing sorghum grain have shown that the analysis of the products is diversified in terms of the examined characteristics. An additional relationship was observed between the content of health-promoting ingredients and the percentage of sorghum in products. At the same time, a similar relationship was found for products containing fruit and seed cover. Whole grain products, including sorghum, are richer in bioactive ingredients due to accumulation in the outer layers of the grain. Based on this research, it was found that the products of processing sorghum and those containing sorghum grains can be classified as functional foods.

## Figures and Tables

**Table 1:** Characteristics of the tested material.

No.	Product	Number of Samples	Composition	Quantity [%]
1.	White sorghum grain flour	4	White sorghum grain	100
2.	Red sorghum grain flour	4	Red sorghum grain	100
3.	Sorghum grain flour	5	Sorghum grain flour	100
4.	Sorghum–dehulled grain	4	Sorghum grain flour	100
5.	Raw pasta	6	Sorghum flour	70
			Pea flour	30
6.	Wafers	5	Grains of sorghum	89.5
			Amaranth	10
			Sea salt	Bd
7.	Cookies	5	Sorghum grain flour	25
			Corn flour	Bd
			Oatmeal	8.3
			Corn starch	Bd

Bd—no data from the manufacturer.

**Table 2:** Antioxidant activity of bioactive compounds (ABTS^•+^ [µmolTROLOX/kg]) and the content of free phenolic acids (FPA [mgGAE/100 g]) in selected food products containing sorghum grains.

No.	Product	ABTS^+●^ (µmol TROLOX/kg)	FPA (mg GAE/100 g)
1.	White sorghum grain flour	885.0 a	689.3 a
2.	Red sorghum grain flour	712.0 a	352.1 b
3.	Sorghum grain flour	649.0 ab	267.5 b
4.	Sorghum–dehulled grain	577.0 b	229.8 b
5.	Raw pasta	385.0 bc	224.1 b
6.	Wafers	412.0 b	256.3 b
7.	Cookies	319.0 c	338.6 b

a, b, c—the same letters in the column mean no significant differences *p* = 0.05.

**Table 3:** Correlation matrix between the analyzed antioxidants, total phenolic acids, and antioxidants activity.

	ABTS^+●^ (µmolTROLOX/kg)	FPA (mg GAE/100 g)	4-Hydroxybenzoic	Caffeic	Chlorogenic	Ferulic	Gallic	*p*-Coumaric	Protocatechuic	Sinapic	Syringic	t-Cinnamic	Vanilic
ABTS^+●^ (µmolTROLOX/kg)	1												
FPA (mg GAE/100 g)	0.704213	1											
4-hydroxybenzoic	**0.845434**	**0.906869**	1										
caffeic	0.609176	0.416305	0.691012	1									
chlorogenic	0.418167	0.071676	0.407603	**0.933454**	1								
ferulic	0.017179	0.591446	0.257984	−0.31636	−0.59464	1							
gallic	0.497809	0.07581	0.417279	**0.90038**	**0.954943**	−0.50838	1						
*p*-coumaric	0.458315	**0.872567**	0.716457	0.117646	−0.18958	0.58202	−0.27464	1					
protocatechuic	**0.791453**	**0.933396**	**0.980841**	0.673062	0.374673	0.385947	0.401756	0.717284	1				
sinapic	0.685558	0.458905	0.709327	**0.986663**	**0.905563**	−0.28554	**0.891183**	0.124897	0.692116	1			
syringic	**0.835824**	**0.975412**	**0.949394**	0.475579	0.149878	0.464739	0.18119	0.81304	**0.94715**	0.528548	1		
t-cinnamic	−0.73116	−0.05505	−0.35099	−0.45557	−0.51652	0.515839	−0.61627	0.113755	−0.25559	−0.50195	−0.25787	1	
vanilic	−0.3558	−0.09258	−0.14326	0.341272	0.383923	0.028883	0.318922	−0.28217	−0.02241	0.320494	−0.22308	0.468929	1

Significant correlation coefficients at the level of 0.95 were marked in bold.

**Table 4:** Content of selected phenolic acids (mg/kg) in food products.

No.	Product	4-Hydroxybenzoic	Caffeic	Chlorogenic	Ferulic	Gallic	*p*-Coumaric	Protocatechuic	Sinapic	Syringic	t-Cinnamic	Vanilic	Total of Phenolic Acid
1.	White sorghum grain flour	26.5 ± 2.34 a	14.25 ± 1.23 b	9.24 ± 1.04 b	205.42 ± 4.84 a	17.25 ± 1.14 bc	117.5 ± 4.72 a	135.6 ± 4.64 a	10.42 ± 0.86 c	18.44 ± 0.96 a	2.66 ± 0.31 c	0.04 ± 0.04 b	557.32 ± 11.12 c
2.	Red sorghum grain flour	14.2 ± 1.26 b	25.85 ± 2.61 a	35.6 ± 3.54 a	89.69 ± 3.75 c	55.25 ± 2.54 a	34.22 ± 2.29 cd	84.5 ± 3.67 b	19.55 ± 1.27 a	6.36 ± 0.13 b	0.52 ± 0.06 d	6.23 ± 0.61 a	371.97 ± 8.17 b
3.	Sorghum grain flour	1.25 ± 0.51 c	2.95 ± 0.72 c	4.26 ± 0.51 c	127.55 ± 4.83 b	11.6 ± 0.26 c	38.55 ± 2.31 c	26.1 ± 1.34 d	2.65 ± 0.52 c	3.74 ± 0.61 b	0.74 ± 0.09 d	0.45 ± 0.03 b	219.88 ± 6.87 a
4.	Sorghum–dehulled grain	4.85 ± 0.71 c	7.25 ± 0.74 bc	10.55 ± 0.86 b	141.25 ± 4.01 b	27.5 ± 1.31 b	27.65 ± 2.12 d	47.52 ± 2.51 c	3.52 ± 0.41 c	2.45 ± 0.31 c	0.16 ± 0.02 d	0.13 ± 0.02 b	272.87 ± 6.98 a
5.	Raw pasta	2.45 ± 0.42 c	5.26 ± 0.61 c	6.74 ± 0.41 c	116.45 ± 3.78 b	9.42 ± 0.86 c	42.52 ± 3.43 c	25.85 ± 1.32 d	1.52 ± 0.61 c	0.88 ± 0.08 c	6.55 ± 0.74 b	1.44 ± 0.11 b	219.61 ± 6.04 a
6.	Wafers	2.16 ± 0.31 c	6.36 ± 0.63 bc	10.25 ± 0.74 b	123.52 ± 3.82 b	10.6 ± 0.74 c	66.36 ± 3.51 b	33.6 ± 1.31 cd	2.15 ± 0.41 c	1.12 ± 0.22 c	4.52 ± 0.14 c	2.15 ± 0.11 b	264.02 ± 7.09 a
7.	Cookies	0.88 ± 0.09 c	3.44 ± 0.31 c	1.25 ± 0.19 c	214.7 ± 4.72 a	6.25 ± 0.51 c	49.16 ± 2.54 c	44.85 ± 2.41 c	1.06 ± 0.91 c	2.65 ± 0.22 c	10.52 ± 0.96 a	6.74 ± 0.48 a	345.83 ± 8.37 b

a, b, c, d—the same letters in the column mean no significant differences *p* = 0.05.

**Table 5:** Content of selected flavonoids (mg/kg) in food products.

No	Product	Apigenin	Kempferol	Luteolin	Naringenin	Quercetin	Rutin	Vitexin	Total Content Flavonoids
1.	White sorghum grain flour	nd	0.12 ± 0.02 a	7.25 ± 1.34 d	2.74 ± 0.51 b	1.52 ± 0.15 b	6.55 ± 0.84 d	1.33 ± 0.14 b	208.51 ± 7.17 d
2.	Red sorghum grain flour	0.85 ± 0.1 a	2.16 ± 0.41 b	0.13 ± 0.02 a	1.25 ± 0.33 b	0.14 ± 0.01 a	0.85 ± 0.07 a	0.11 ± 0.01 a	16,73 ± 1.07 a
3.	Sorghum grain flour	0.66 ± 0.08 a	0.41 ± 0.03 a	2.32 ± 0.33 b	1.12 ± 0.24 b	0.49 ± 0.02 a	0.16 ± 0.02 a	0.27 ± 0.01 a	21.97 ± 0.92 a
4.	Sorghum–dehulled grain	0.24 ± 0.02 a	1.23 ± 0.35 b	4.36 ± 0.61 c	0.74 ± 0.12 a	0.28 ± 0.02 a	3.47 ± 0.67 c	0.11 ± 0.01 a	15.31 ± 0.88 a
5.	Raw pasta	0.35 ± 0.04 a	0.28 ± 0.03 a	2.52 ± 0.28 b	0.36 ± 0.02 a	0.34 ± 0.01 a	2.46 ± 0.44 b	0.19 ± 0.01 a	10.62 ± 0.81 a
6.	Wafers	0.56 ± 0.02 a	0.16 ± 0.02 a	0.33 ± 0.02 a	0.66 ± 0.03 a	0.19 ± 0.01 a	0.33 ± 0.04 a	0.15 ± 0.01 a	8.74 ± 0.76 b
7.	Cookies	0.12 ± 0.01 a	0.37 ± 0.03 a	0.17 ± 0.01 a	0.47 ± 0.03 a	0.17 ± 0.01 a	0.27 ± 0.04 a	0.14 ± 0.01 a	4.83 ± 0.36 b

a, b, c, d—the same letters in the column mean no significant differences *p* = 0.05; nd—no data.

**Table 6:** Content of selected phytosterols (mg/kg) in food products.

No.	Product	Beta-Sitosterol	Campesterol	Stigmasterol	Total Content Phytosterols
1.	White sorghum grain flour	29.14 ± 1.47 a	7.32 ± 0.31 a	6.16 ± 0.41 a	42.62 ± 3.57 c
2.	Red sorghum grain flour	12.33 ± 0.31 b	5.84 ± 0.25 a. b	4.05 ± 0.33 b	22.22 ± 1.22 b
3.	Sorghum grain flour	9.52 ± 0.26 b	4.09 ± 0.21 b	5.08 ± 0.76 a	18.69 ± 1.03 b
4.	Sorghum–dehulled grain	2.85 ± 0.13 c	4.33 ± 0.19 b	3.66 ± 0.51 b	10.84 ± 0.89 b
5.	Raw pasta	4.08 ± 0.16 c	1.07 ± 0.08 c	0.45 ± 0.02 c	5.6 ± 0.62 a
6.	Wafers	3.52 ± 0.11 c	1.32 ± 0.09 c	0.36 ± 0.01 c	5.2 ± 0.54 a
7.	Cookies	2.77 ± 0.12 c	2.00 ± 0.09 c	1.16 ± 0.08 c	5.93 ± 0.49 a

a, b, c—the same letters in the column mean no significant differences *p* = 0.05.

## Data Availability

Poznań University of Life Sciences, Faculty of Forestry and Wood Technology, the Department of Chemistry, ul. Wojska Polskiego 75, 60-101 Poznań, Poland.
